# Prostacyclin-producing human mesenchymal cells target H19 lncRNA to augment endogenous progenitor function in hindlimb ischaemia

**DOI:** 10.1038/ncomms11276

**Published:** 2016-04-15

**Authors:** Yuxiao Deng, Zhongwei Yang, Toya Terry, Su Pan, Darren G. Woodside, Jingxiong Wang, Kehe Ruan, James T. Willerson, Richard A. F. Dixon, Qi Liu

**Affiliations:** 1 Wafic Said Molecular Cardiology Research Laboratory, Texas Heart Institute, P.O. Box 20345, MC 2-255, Houston, Texas 77225-0345, USA; 2 Department of Pharmacological and Pharmaceutical Sciences, University of Houston, 552 Science and Research Building 2, Houston, Texas 77204-5037, USA

## Abstract

Promoting the paracrine effects of human mesenchymal stem cell (hMSC) therapy may contribute to improvements in patient outcomes. Here we develop an innovative strategy to enhance the paracrine effects of hMSCs. In a mouse hindlimb ischaemia model, we examine the effects of hMSCs in which a novel triple-catalytic enzyme is introduced to stably produce prostacyclin (PGI_2_-hMSCs). We show that PGI_2_-hMSCs facilitate perfusion recovery and enhance running capability as compared with control hMSCs or iloprost (a stable PGI_2_ analogue). Transplanted PGI_2_-hMSCs do not incorporate long term into host tissue, but rather they mediate host regeneration and muscle mass gain in a paracrine manner. Mechanistically, this involves long noncoding RNA H19 in promoting PGI_2_-hMSC-associated survival and proliferation of host progenitor cells under hypoxic conditions. Together, our data reveal the novel ability of PGI_2_-hMSCs to stimulate host regenerative processes and improve physical function by regulating long noncoding RNA in resident progenitor cells.

## Abstract (web summary)

Human mesenchymal stem cells (hMSC) expressing paracrine factors may enhance therapeutic benefits when transplanted. Here, the authors show that hMSCs stably expressing prostacyclin enhance host regeneration and muscle mass gain in a mouse hindlimb ischaemia model, mediated by the long noncoding RNA H19.

## Graphical abstract

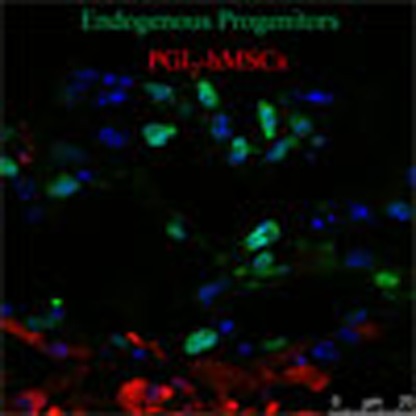

Growth factors, cytokines, short noncoding RNAs and exosomes that are released from human mesenchymal stem cells (hMSCs) are important paracrine components in the positive outcome of cell therapies^1^. These elements contribute to host regenerative processes and facilitate tissue repair/regeneration^2^. Efforts are ongoing to characterize these paracrine components^3^. However, a major challenge in developing therapeutic regenerative strategies based on these elements is mimicking their temporal and spatial release from transplanted hMSCs under local pathological conditions. Moreover, since long-term engraftment of transplanted hMSCs is minimal, therapeutic benefits may be maximized by enhancing the paracrine effects of hMSCs early after cell transplantation to increase the host regenerative or protective responses to tissue damage. Thus, we have modified hMSCs with the intention of improving their paracrine ability to protect endogenous progenitor cells under ischaemic conditions, as ischaemia is the most common clinical condition leading to cell damage^4^.

We and others have shown that prostacyclin (PGI_2_) is a key instructive molecule for promoting angiogenesis and revascularization^5^,^6^. Sustained delivery of a PGI_2_ stable analogue into ischaemic hindlimbs stimulated the simultaneous secretion of chemokines and other soluble factors from ischaemic muscle, resulting in enhanced perfusion recovery and vascular growth^5^. These findings indicate that PGI_2_ acts as a master regulator to control the coordinated secretion of multiple elements from cells. However, targeted delivery of PGI_2_ into ischaemic tissue is challenging because of the instability of the molecule. Thus, developing a biological carrier or generator of PGI_2_ would facilitate the direct delivery of PGI_2_ to ischaemic tissues or the production of PGI_2_ at the site of tissue injury. To this end, we have produced hMSCs that stably secrete PGI_2_ (PGI_2_-hMSCs). We speculate that the consistent release of PGI_2_ from PGI_2_-hMSCs in target tissues may not only overcome the current inconvenience of PGI_2_ delivery but also enhance the overall paracrine effects of hMSCs through the synergistic actions of PGI_2_ and other biochemical mediators released from cells.

In the current study, we have engineered hMSCs to stably produce PGI_2_ (PGI_2_-hMSCs) and tested their effects on perfusion recovery, exercise capacity and muscle mass build-up in a mouse hindlimb ischaemia model. We have observed significant increases in these variables with PGI_2_-hMSC therapy as compared with hMSC alone and iloprost (ILO, a stable PGI_2_ analogue) alone. PGI_2_-hMSC therapy is associated with the cytoprotective effects within endogenous tissue resident progenitor cells that mechanistically involved the long noncoding RNA (lncRNAs) H19 as a critical element for cell survival/proliferation. Our results shed light on the potential clinical application of PGI_2_-hMSCs in treating cardiovascular diseases.

## Results

### PGI_2_-hMSCs improve blood perfusion and running endurance

**Figure 1:**
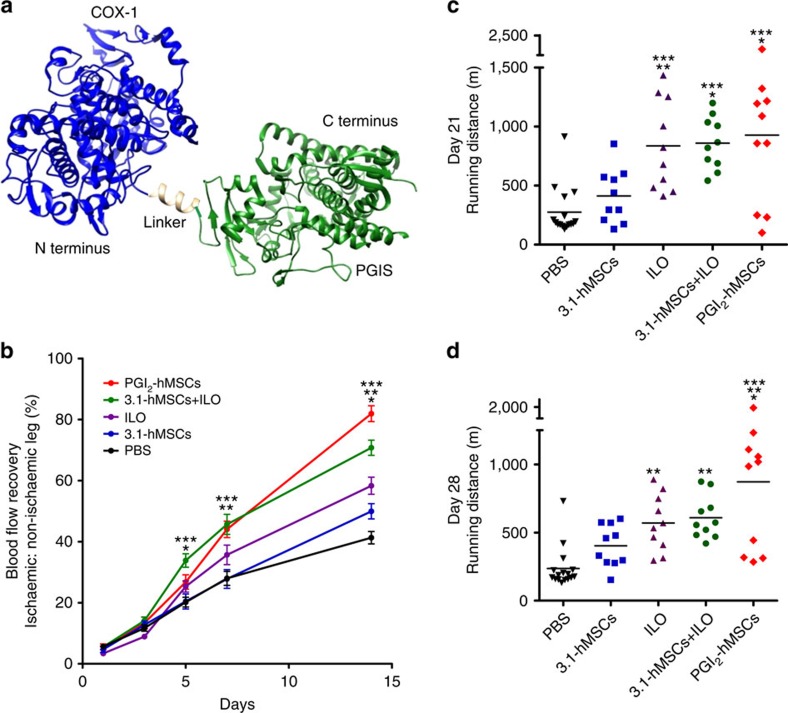
PGI_2_-hMSC therapy concurrently improved distal perfusion and treadmill performance after hindlimb ischaemia. (**a**) Schematic cartoon illustration of the COX-1-10aa-PGIS fusion protein. A His–Ala–Ile–Met–Gly–Val–Ala–Phe–Thr–Trp peptide linker (from a helical transmembrane domain of bovine rhodopsin) was used to connect the chimeric protein and to maintain the topology of both COX-1 and PGIS proteins. Structures used for generation of the schematic included PDB 3N8Z (COX-1)^33^, PDB 2IAG (PGIS)^34^ and PDB 1GZM (rhodopsin)^35^. (**b**) Quantitative analysis of perfusion from ankle to toe showed different rates of blood flow recovery among the five treatment groups over the 14-day observation period. Notably, at day 14 after cell administration, perfusion in the PGI_2_-hMSC group exceeded that in the four other treatment groups, leading to significantly better perfusion recovery. Day 5: *P<0.05 for 3.1-hMSCs+ILO versus ILO or versus PGI_2_-hMSCs; ***P<0.001 for 3.1-hMSCs+ILO versus 3.1-hMSCs or versus PBS. Day 7: **P<0.01 for 3.1-hMSCs+ILO versus 3.1-hMSCs; PGI_2_-hMSCs versus 3.1-hMSCs. ***P<0.001 for 3.1-hMSCs+ILO versus PBS; PGI_2_-hMSCs versus PBS. Day 14, *P<0.05 for 3.1-hMSCs versus PBS, ILO versus 3.1-hMSCs; **P<0.01 for 3.1-hMSCs+ILO versus ILO, PGI_2_-hMSCs versus 3.1-hMSCs+ILO; ***P<0.001 for ILO versus PBS, 3.1-hMSCs+ILO versus PBS or versus 3.1-hMSCs, PGI_2_-hMSCs versus PBS or versus ILO or versus 3.1-hMSCs. (**c**) In run-to-exhaustion tests, mice treated with PGI_2_-hMSCs, daily injections of ILO or 3.1-hMSCs+ILO had a significantly longer maximal running distance than those treated with 3.1-hMSCs or vehicle (PBS) at 21 days. *P<0.05 for PGI_2_-hMSCs versus 3.1-hMSCs; 3.1-hMSCs+ILO versus 3.1-hMSCs. **P<0.01 for ILO versus 3.1-hMSCs; ***P<0.001 for ILO versus PBS; 3.1-hMSCs+ILO versus PBS; PGI_2_-hMSCs versus PBS. (**d**) At 28 days, the performance enhancement in PGI_2_-hMSC-treated mice was similar to that seen at day 21. Meanwhile, 3.1-hMSCs+ILO outperformed ILO treatment when compared with 3.1-hMSC treatment (one-way ANOVA followed by Dunnett's multiple comparison test). *P<0.05 for PGI_2_-hMSCs versus ILO or versus 3.1-hMSCs+ILO. **P<0.01 for ILO versus PBS, 3.1-hMSCs+ILO versus PBS, PGI_2_-hMSCs versus 3.1-hMSCs. ***P<0.001 for PGI_2_-hMSCs versus PBS. Statistical significance was determined by one-way ANOVA followed by Newman–Keuls *post hoc* test. Data are shown as mean±s.e.m. *N*=16 of sex-matched mice in vehicle (PBS) group; *N*=10 sex-matched mice each in the other four treatment groups.

We created PGI_2_-overexpressing hMSCs by introducing an active triple-catalytic enzyme that links cyclooxygenase-1 to prostacyclin synthase (COX-1-10aa-PGIS) based on our previous biochemical and structural studies of COX-1 and PGIS (Fig. 1a)^7^. This triple-catalytic enzyme catalyses three key reactions that allow the production of PGI_2_ from arachidonic acid as previously described^8^. We confirmed the stable expression of the transgene in PGI_2_-hMSCs by genomic PCR and western blot (a 130-kDa protein; Supplementary Fig. 1a,b). To assess the production of PGI_2_, we used an enzyme immunoassay to measure the metabolite 6-keto PGF1α. Compared with hMSCs (native hMSCs containing no vector) and 3.1-hMSCs (hMSCs containing pcDNA3.1 [vector used to construct pcDNA COX-1-10aa-PGIS]), PGI_2_-hMSCs were capable of secreting a fivefold higher concentration of 6-keto PGF1α in the supernatant after incubating cells with arachidonic acid for 20 min (Supplementary Fig. 1c; *P*<0.01; one-way analysis of variance [ANOVA]).

We examined the efficacy of PGI_2_-hMSCs (4.5 × 10^5^ cells per mouse, single intramuscular injection) in restoring blood flow in mouse ischaemic hindlimbs. Sex- and age-matched non-obese diabetic–severe combined immunodeficient (NOD–SCID) mice received one of the following treatments after undergoing the same surgical procedures: (1) vehicle (PBS); (2) a single intramuscular injection of 3.1-hMSCs (4.5 × 10^5^ cells per mouse); (3) daily injections with ILO, a stable PGI_2_ analogue (2 ng per kg per day, intraperitoneally), for 21 days; (4) a single intramuscular injection of 3.1-hMSCs (4.5 × 10^5^ cells per mouse) accompanied by daily injections of ILO (2 ng per kg per day, intraperitoneally) for 21 days (3.1-hMSCs+ILO); and (5) a single intramuscular injection of PGI_2_-hMSCs (4.5 × 10^5^ cells per mouse). Perfusion was quantified 24 h after treatment and up to 14 days thereafter. We focused our measurements on regional perfusion from ankle to toe because these lower hindlimb extremities are most affected by the obstruction of the femoral artery. Although perfusion to ischaemic feet increased in all the groups over the 14-day measurement period, the rates of recovery in response to various treatments differed (Fig. 1b). In the initial 3 days, perfusion was similar regardless of treatment. At day 5, perfusion in 3.1-hMSCs+ILO-treated mice exceeded that in all the groups (Fig. 1b). Intriguingly, mice started to respond quickly to PGI_2_-hMSC therapy after day 5. By 7 days, perfusion was significantly (as determined by one-way ANOVA) better in mice treated with PGI_2_-hMSCs than in those treated with either 3.1-hMSCs or PBS (Fig. 1b), whereas perfusion in mice treated with the 3.1-hMSCs+ILO combination also remained significantly better than that in mice treated with 3.1-hMSCs or PBS (Fig. 1b). At day 14, perfusion in mice treated with PGI_2_-hMSCs significantly exceeded that in those treated with the combination of 3.1-hMSCs+ILO (81.93±2.59% versus 70.79±2.48%, *P*<0.01; one-way ANOVA), leading to the most marked recovery of perfusion in ischaemic lower extremities among the five groups. Perfusion at day 14 was also significantly better in 3.1-hMSCs+ILO-treated mice than in mice treated with vehicle, ILO or 3.1-hMSCs. By the end at 14 day, the order of treatment efficacy was PGI_2_-hMSCs >3.1-hMSCs+ILO>ILO>3.1-hMSCs>PBS.

To assess whether the improvement of blood perfusion in PGI_2_-hMSC therapy concurrently increases muscle strength and functional ability long term, we used a run-to-exhaustion performance test. At 21 days after cell delivery, PGI_2_-hMSC-treated mice ran more than twice the distance of 3.1-hMSC-treated mice (926.94±196.33 versus 412.15±73.06 m; *P*<0.05; one-way ANOVA; Fig. 1c). The average running distance between 3.1-hMSCs+ILO-treated and ILO-treated mice was comparable and was twice as long as that in 3.1-hMSC-treated mice (Fig. 1c). Mice treated with PGI_2_-hMSCs, 3.1-hMSCs+ILO or ILO all performed significantly better than mice treated with PBS (one-way ANOVA; Fig. 1c).

To test whether the daily administration of ILO acutely affects running endurance, we discontinued ILO at day 21 and repeated the forced running experiments at day 28. Running endurance declined in both 3.1-hMSCs+ILO- (608.74±49.63 m at day 28; Fig. 1d) and ILO-treated mice (570.00±65.35 m) as compared with that at day 21, respectively, indicating that continued dosing of ILO is needed to maintain similar improved performance. Although discontinuing ILO reduced running distance at day 28, the endurance benefit of 3.1-hMSCs+ILO treatment over ILO treatment was evident when the two groups were compared with 3.1-hMSC treatment; 3.1-hMSCs+ILO treatment, but not ILO treatment, showed sustained significant benefits over 3.1-hMSC treatment at 28 days (*P*<0.05; one-way ANOVA with Dunnett's multiple comparison test; Fig. 1d). The exercise ability of PGI_2_-hMSC-treated mice was similar at days 21 and 28, remaining significantly better than that of 3.1-hMSC-treated mice at day 28 (871.40±169.04 versus 402.44±48.93 m; *P*<0.01; one-way ANOVA; Fig. 1d). The overall performance was PGI_2_-hMSCs >3.1-hMSCs+ILO combination >ILO>3.1-hMSCs>PBS. Together, our perfusion and treadmill performance tests suggest PGI_2_-hMSC treatment is superior to vehicle, 3.1-hMSC, ILO and 3.1-hMSCs+ILO treatment in improving functional recovery in ischaemic hindlimbs.

### PGI_2_-MSCs show enhanced acute retention in ischaemic legs

**Figure 2:**
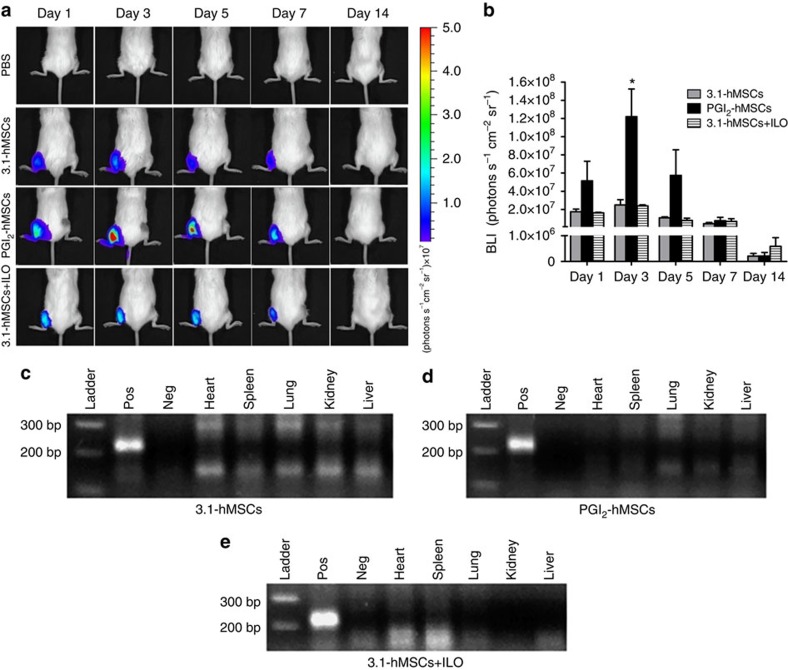
PGI_2_-hMSCs showed superior acute retention in ischaemic hindlimbs. (**a**) Representative *in vivo* bioluminescent images of NOD–SCID mice at indicated intervals after injection of vehicle (PBS), 3.1-hMSCs, PGI_2_-hMSCs or 3.1-hMSCs+ILO into the gastrocnemius muscle of the ischaemic hindlimbs. The minimal noninvasive visualized value was 1 × 10^6^ photons s^−1^ cm^−2^ sr^−1^. (**b**) Quantification of maximal bioluminescence in cell-treated ischaemic hindlimbs at the indicated time. *P<0.05, PGI_2_-hMSCs versus 3.1-hMSCs or versus 3.1-hMSCs+ILO by a one-way ANOVA with Newman–Keuls *post hoc* test. Data are shown as mean±s.e.m. *N*=5 mice (male and female) per treatment group. All treatment groups were sex matched. (**c**–**e**) hMSCs were not detected in the heart, spleen, lung, kidney or liver at 14 days after cell delivery.

The benefit of PGI_2_-hMSC therapy could stem from their long-term superior engraftment in ischaemic hindlimbs when compared with 3.1-hMSCs. To track the fate of exogenously delivered cells longitudinally, we transduced hMSCs with a lentiviral construct that contains the triple-fusion reporter genes of human herpes simplex virus type-1-thymidine kinase, mCherry fluorophore and firefly luciferase (Supplementary Fig. 2). Luciferase catalyses light-emitting photochemical reactions of luciferin in live cells, which enables whole-body imaging to track the distribution and engraftment of transplanted cells. We performed a lentiviral infection of hMSCs 3 days before producing PGI_2_-hMSCs and 3.1-hMSCs, in parallel, to ensure that the lentiviral transduction efficiency was the same in both cell types. *In vitro* luciferase assays with lentivirus-infected hMSCs indicated that bioluminescent intensity readings correlated positively with cell numbers (Supplementary Fig. 2). Immediately after injecting luciferin into the mice, we recorded sequential readings until the maximal signal intensity was reached. During the measuring time after cell administration, whole-body images showed that bioluminescence was seen only in ischaemic hindlimbs. No signals above background level were detected in other organs/tissues or in vehicle-treated mice (Fig. 2a). At day 1 after cell injection, we found a higher maximal bioluminescent intensity in the ischaemic hindlimbs of PGI_2_-hMSC-treated mice compared with those that received 3.1-hMSCs or 3.1-hMSCs+ILO (5.14±2.16 × 10^7^ versus 1.75±0.29 × 10^7^ or 1.63±0.50 × 10^7^ photons s^−1^ cm^−2^ sr^−1^, respectively; Fig. 2b). The bioluminescent signal in the PGI_2_-hMSC-treated group peaked at day 3 (12.20±3.05 × 10^7^ photons s^−1^ cm^−2^ sr^−1^) and was significantly higher than that seen in mice treated with 3.1-hMSCs or 3.1-hMSCs+ILO (2.49±0.58 × 10^7^ and 2.40±1.10 × 10^7^ photons s^−1^ cm^−2^ sr^−1^, respectively; *P*<0.05; one-way ANOVA). The signal from the PGI_2_-hMSC-treated group began to decrease by day 5 (5.74±2.82 × 10^7^ versus 1.08±0.11 × 10^7^ or versus 0.80±0.24 × 10^7^ photons s^−1^ cm^−2^ sr^−1^ in PGI_2_-hMSC, 3.1-hMSC and 3.1-hMSCs+ILO-treated mice, respectively). At day 14, we detected a large reduction in the signal to close to background levels (1.00 × 10^5^ photons s^−1^ cm^−2^ sr^−1^) in the readings in each group. This exponential decline of bioluminescence indicated the sharp loss of hMSCs at 14 days post injection.

We examined other organ tissue for the presence of hMSCs at 14 days after cell injections into ischaemic legs. We used human-specific primers for the *HLA-A* gene^9^ to perform PCR on genomic DNA isolated from the heart, spleen, lung, kidney and liver of mice treated with 3.1-hMSCs, 3.1-hMSCs+ILO and PGI_2_-hMSCs. Genomic DNA of hMSCs (20 ng) mixed with genomic DNA from the gastrocnemius muscle of vehicle-treated mice was used as a positive control. In parallel PCR analyses, we detected no human-specific PCR product (214 bp) in any organ examined (except for the positive control; Fig. 2c-e), suggesting that hMSCs did not migrate to other organs at or after 14 days. Overall, although the acute retention of PGI_2_-hMSCs was superior to that of 3.1-hMSCs or 3.1-hMSCs+ILO, the limited long-term (after day 14) presence of PGI_2_-hMSCs suggests that it is unlikely that they participate directly in tissue regeneration/repair through permanent engraftment.

### PGI_2_-hMSCs increase muscle mass of ischaemic legs

**Figure 3:**
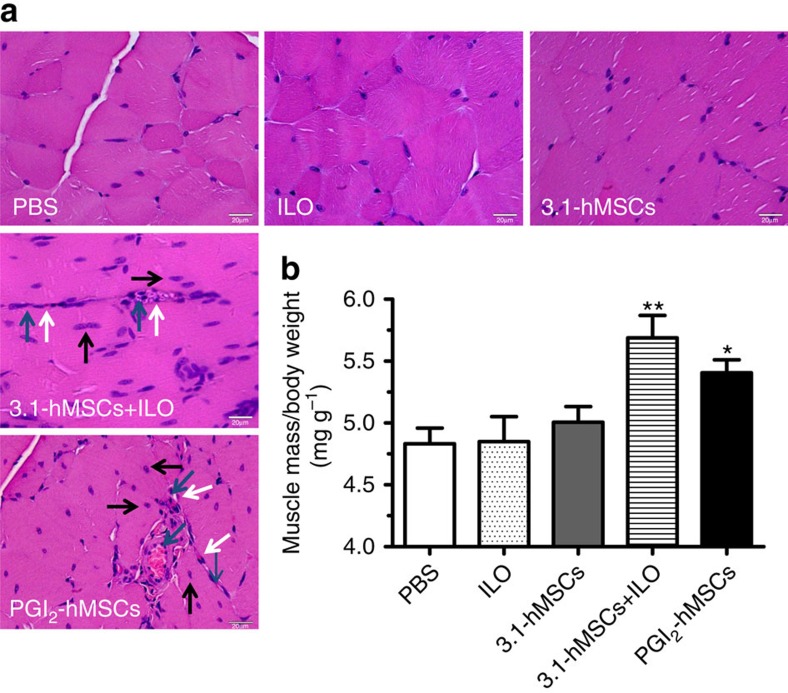
PGI_2_-hMSC treatment led to host regeneration and muscle mass gain. (**a**) Haematoxylin and eosin (H&E) stain showed regenerating myofibers with centralized nuclei (black arrows) next to microvessels (white arrows) containing red blood cells (blue arrows) at day 14 in mice treated with 3.1-hMSCs+ILO or PGI_2_-hMSCs. (**b**) Muscle mass relative to body weight. Both 3.1-hMSCs+ILO- and PGI_2_-hMSC-treated mice had significant muscle mass gain in the gastrocnemius muscle in ischaemic hindlimbs as compared with the three other treatments after 1 month. **P<0.01, 3.1-hMSCs+ILO versus ILO, versus 3.1-hMSCs or versus PBS; *P<0.05, PGI_2_-hMSCs versus ILO, versus 3.1-hMSCs or versus PBS by a one-way ANOVA with Newman–Keuls *post hoc* test. Data are shown as mean±s.e.m. *N*=9 mice/PBS; *N*=5 mice/ILO; *N*=10 mice/3.1-hMSCs; *N*=10 mice/3.1-hMSCs+ILO; *N*=10 mice/PGI_2_-hMSCs. Both male and female mice were used. Scale bar, 20  μm.

Because the improved treadmill running of mice that received PGI_2_-hMSCs was not due to long-term exogenous cell engraftment/differentiation in ischaemic hindlimbs, we studied how ischaemic muscle reacts to the treatment regimen by performing histological examinations. Histological examination of gastrocnemius muscle sections of ischaemic hindlimbs at 14 days after cell injections showed host muscle regeneration in PGI_2_-hMSC-treated mice (Fig. 3a) rather than cell engraftment. The newly formed myofibers had centralized nuclei and were located close to arterioles or capillaries (Fig. 3a). The presence of red blood cells within the arterioles or capillaries indicated the perfusion of oxygenated blood in the regenerated region. We also noted areas of new myofiber formation in the sections from mice treated with 3.1-hMSCs+ILO; however, few newly formed myofibers were seen with ILO, 3.1-hMSC or vehicle treatment (Fig. 3a). These findings suggest that exogenous PGI2-hMSCs or 3.1-hMSCs+ILO promoted endogenous regeneration in the gastrocnemius muscle of ischaemic hindlimbs (Fig. 3a).

Because regeneration may lead to muscle growth, we measured the mass of the gastrocnemius muscle in legs 1 month after treatment. The absolute muscle mass varies among mice, so we evaluated muscle growth by measuring the gastrocnemius muscle mass relative to body weight. Muscle mass significantly increased (by 8–12%) in PGI_2_-hMSC-treated mice as compared with that in mice treated with 3.1-hMSC, ILO or vehicle (*P*<0.05; one-way ANOVA; Fig. 3b). A gain in muscle mass was also seen in 3.1-hMSCs+ILO treatment as compared with 3.1-hMSC, ILO and vehicle treatment (*P*<0.01; one-way ANOVA; Fig. 3b). No significant difference was found between PGI_2_-hMSC and 3.1-hMSC+ILO treatment. These findings suggest that the enhanced performance associated with PGI_2_-hMSC therapy may be due, in part, to the paracrine effects of PGI_2_-hMSCs on endogenous progenitor cells resulting in muscle regeneration.

### Sca-1^+^Ki67^+^ cells accumulate at PGI_2_-hMSC injection sites

**Figure 4:**
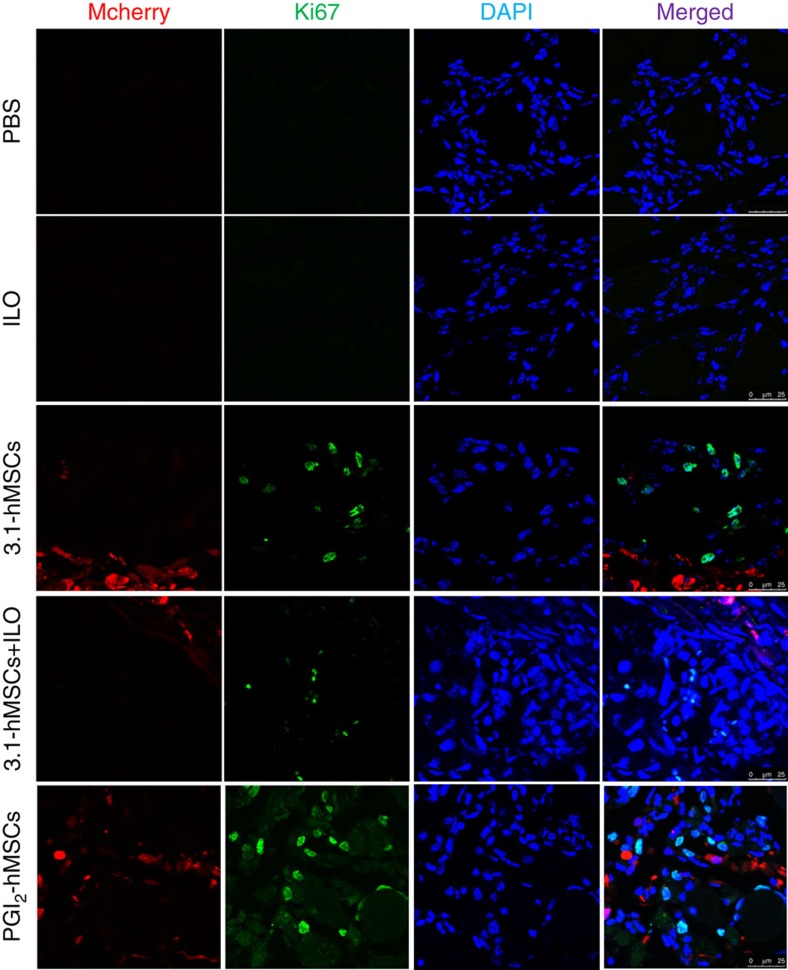
Human mesenchymal stem cells mediated host cell proliferation. Representative immunofluorescence confocal images illustrate endogenous Ki67^+^ cells spread within the hMSC injection area at day 3. Human mesenchymal cells contain red fluorescent mCherry protein. Ki67^+^ cells were rarely seen in the gastrocnemius muscles of ischaemic legs of vehicle- and ILO-treated mice. Scale bar, 25 μm.

**Figure 5:**
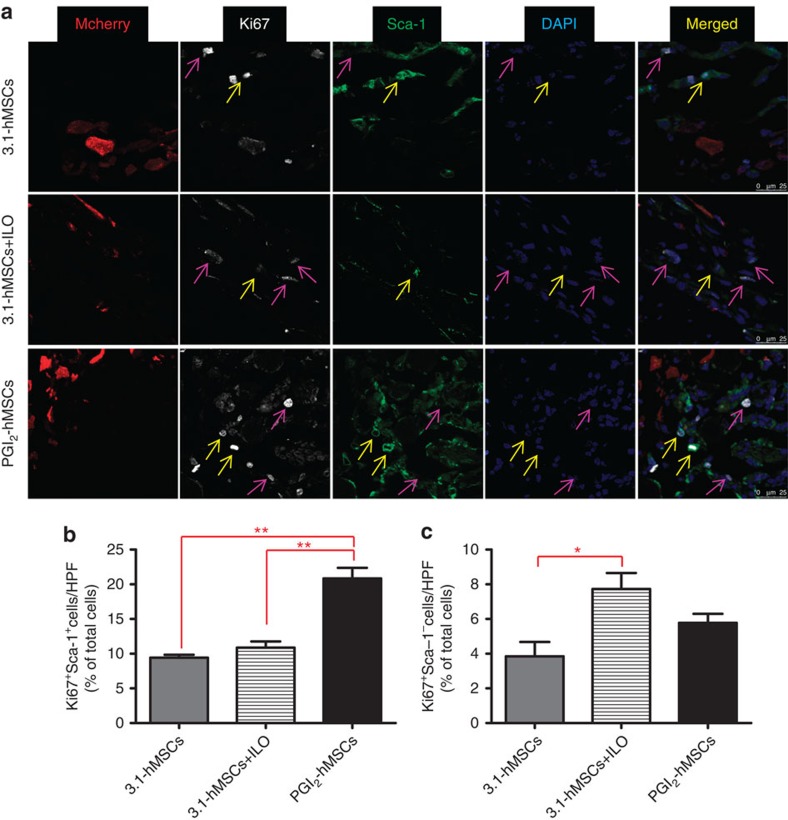
Significantly higher numbers of endogenous proliferating Ki67^+^Sca-1^+^ cells were located adjacent to the PGI_2_-hMSC site 3 days after cell injections. (**a**) Representative confocal images illustrate the distribution of endogenous Ki67^+^Sca-1^+^ (yellow arrows) and Ki67^+^Sca-1^−^cells (purple arrows) in ischaemic gastrocnemius muscle areas surrounding injected hMSCs. (**b**) Quantitative analysis indicated a significantly higher percentage of Ki67^+^Sca-1^+^cells surrounding PGI_2_-hMSCs injection sites as compared with 3.1-MSCs+ILO or to 3.1-hMSCs sites (**P<0.01; one-way ANOVA with Newman–Keuls *post hoc* test). (**c**) Significantly higher percentage of Ki67^+^Sca-1^−^cells surrounded 3.1-MSCs+ILO injection sites than 3.1-hMSC injection sites (*P<0.05; one-way ANOVA with Newman–Keuls *post hoc* test). No significant differences were seen between 3.1-MSCs+ILO and PGI_2_-hMSC treatment or between PGI_2_-hMSC and 3.1-hMSC treatment. Data are shown as mean±s.e.m. A total of 30 high-power fields (HPFs) from three mice (male and female) per treatment group (10 HPFs per mouse, see details in Method) was used. Mice were sex matched among the three treatment groups. Scale bar, 25 μm.

To determine whether PGI_2_-hMSCs induced an endogenous cellular response, we examined their effects *in situ* during hindlimb ischaemia. Immunofluorescence staining of gastrocnemius muscle obtained 3 days after cell delivery showed the presence of cells positive for the proliferation-associated nuclear protein Ki67. Ki67^+^ cells tended to localize in areas adjacent to the location of PGI_2_-hMSCs (Fig. 4) and were rarely observed in regions further away from PGI_2_-hMSCs (>250 μm distance). Notably, most Ki67^+^ cells expressed stem cell antigen-1 (Sca-1; Fig. 5a), a common marker on a variety of stem/progenitor cells, including haematopoietic cells, mesenchymal stem cells, endothelial progenitor cells (EPCs) and a muscle side population of stem cells^10^,^11^,^12^,^13^,^14^. In addition, we found a similar anatomical distribution of endogenous Ki67^+^ cells in 3.1-hMSCs+ILO- and 3.1-hMSC-treated mice. When we quantified cell numbers in areas surrounding injected hMSCs (125 × 125 μm^2^), the accumulation of Ki67^+^Sca-1^+^cells was about twofold higher in PGI_2_-hMSC-treated mice than in mice treated with 3.1-hMSCs+ILO or 3.1-hMSCs (*P*<0.01; one-way ANOVA; Fig. 5b). Because Sca-1^−^ cell populations contain satellite cells that contribute to muscle regeneration^15^, we quantified Ki67^+^Sca-1^−^cells in the same samples. In contrast to our results with Ki67^+^Sca-1^+^ cells, we found significantly higher numbers of Ki67^+^Sca-1^−^cells at 3.1-hMSCs+ILO injection sites than at 3.1-hMSC sites (*P*<0.05; one-way ANOVA; Fig. 5c). Thus, injections of PGI_2_-hMSCs and 3.1-hMSCs+ILO helped to create a focal area to foster proliferation of endogenous progenitor cells that led to muscle mass gain.

### Multiple paracrine factors are released from PGI_2_-hMSCs

**Figure 6:**
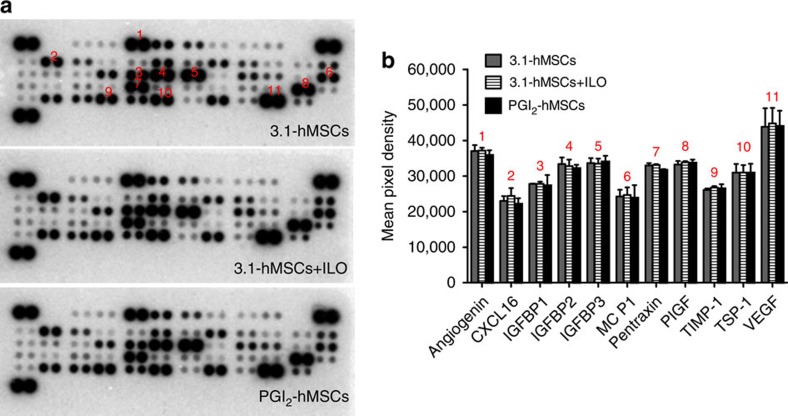
PGI_2_-hMSCs did not affect the natural ability of hMSCs to release pro-survival and pro-angiogenic factors under hypoxia. (**a**) Representative images of proteome profiler arrays probed with 3.1-hMSC-conditioned medium (CM), 3.1-hMSCs+ILO-CM, or PGI_2_-hMSC-CM. (**b**) Analysis of the differential expression of angiogenic factors and chemokines from three independent arrays. CM collected from 3.1-hMSCs, 3.1-hMSCs+ILO or PGI_2_-hMSCs contained similar levels of soluble proteins known to promote survival and angiogenesis under ischaemic conditions. Data are shown as mean±s.e.m. from three independent arrays. CXCL16, chemokine (C–X–C motif) ligand 16; IGFBP1-3, insulin-like growth factor-binding protein 1-3; MCP-1, monocyte chemoattractant protein-1; PIGF, placental growth factor; TIMP-1, TIMP metallopeptidase inhibitor 1; TSP-1, thrombospondin-1; VEGF, vascular endothelial growth factor.

Given that native hMSCs exert pro-survival effects under ischaemia by secreting paracrine factors, we assessed whether the release of high levels of PGI_2_ from PGI_2_-hMSCs or the incubation of 3.1-hMSCs with ILO affects the secretion pattern of soluble proteins from hMSCs. Thus, we performed a proteome profiler array with conditioned media collected from cultures of PGI_2_-hMSCs, 3.1-hMSCs+ILO and 3.1-hMSCs that were grown without serum under hypoxic conditions (1.5% O_2_) mimicking the low-oxygen tension seen in ischaemic hindlimbs. We compared the simultaneous basal secretion of 54 soluble factors. The relative amounts of most factors in the conditioned medium of PGI_2_-hMSC and 3.1-hMSCs+ILO cultures were comparable to the levels from 3.1-hMSC cultures (Fig. 6a). Only a few factors showed subtle variations among the groups, and those results are given in Fig. 6b. Our findings suggest that PGI_2_ or ILO did not interfere with the natural ability of hMSCs to release high levels of cytoprotective, pro-survival and pro-angiogenic factors.

### PGI_2_-hMSCs provide myoblast cytoprotection via H19 lncRNA

**Figure 7:**
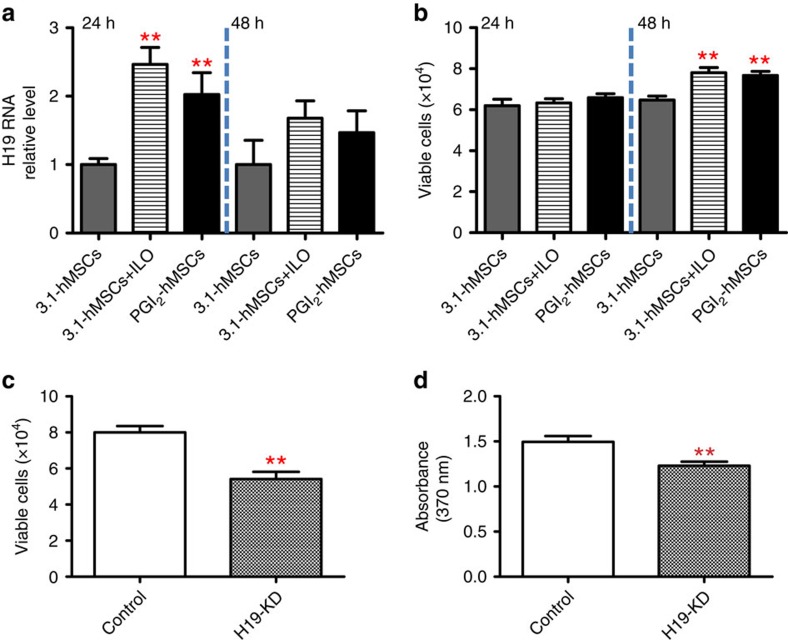
H19 lncRNA promoted host myoblasts survival and proliferation under hypoxia (1.5% O_2_). (**a**) Primary myoblasts co-cultured with PGI_2_-hMSCs or with 3.1-hMSCs+ILO for 24 h showed a significant increase in H19 lncRNA levels as compared with those co-cultured with 3.1-hMSCs. The increase in H19 lncRNA levels was transient; at 48 h, levels were comparable among all the groups. (**b**) No differences were seen in the total number of viable cells among myoblasts co-cultured with PGI_2_-hMSCs, 3.1-hMSCs+ILO or 3.1-hMSCs for 24 h. However, by 48 h, the number of viable myoblasts was significantly higher when cells were co-cultured with PGI_2_-hMSCs or 3.1-hMSCs+ILO than with 3.1-hMSCs. (**c**) *H19* silencing reduced the paracrine effects of PGI_2_-hMSCs on myoblast survival. The total number of viable cells was significantly reduced in myoblasts with H19 deficiency (H19 KD) that were co-cultured with PGI_2_-hMSCs for 48 h as compared with control myoblasts co-cultured with PGI_2_-hMSCs in parallel. (**d**) The paracrine effects of PGI_2_-hMSCs on myoblast proliferation were also impaired with *H19* silencing. Myoblasts with H19 deficiency that were co-cultured with PGI_2_-hMSCs for 48 h showed a significant reduction in cell proliferation as compared with control myoblasts co-cultured in parallel with PGI_2_-hMSCs. **P<0.01. Statistical significance was determined by one-way ANOVA with Newman–Keuls *post hoc* test (**a**,**b**) and a two-tailed *t*-test (**c**,**d**). Data are shown as mean±s.e.m. from three to four replicates and are representative of three independent experiments with similar results.

To gain insight into the endogenous regenerative capability mediated by PGI_2_-hMSCs in a hostile microenvironment, we conducted *in vitro* co-culture mechanistic studies under hypoxia (1.5% O_2_). Myogenic progenitor cells (myoblasts) were used to assess the paracrine effects of PGI_2_-hMSCs because we observed muscle regeneration at day 14 and muscle mass gain at day 30 in PGI_2_-hMSC-treated mice. We chose to use transwell co-culturing of PGI_2_-hMSCs (resulting in a constant release of PGI_2_) and myoblasts rather than conditioned media from PGI_2_-hMSCs to study the paracrine effects because PGI_2_ is not stable, with a half-life of 1–2 min (ref. 16). Emerging evidence has shown that long noncoding RNAs contribute significantly to diverse biological functions such as cell proliferation, survival and differentiation^17^. Specifically, H19 lncRNA has been identified as an important factor in regulating skeletal muscle development^18^,^19^. Dey *et al*. showed that H19 lncRNA encodes miR-675-3p and miR-675-5p, which stimulate myogenesis. H19 deficiency in skeletal muscle resulted in impaired regeneration and smaller myofiber size after injury^18^. Thus, we sought to determine whether H19 lncRNA was involved in PGI_2_-hMSC-induced myogenesis. To this end, we first assessed whether PGI_2_-hMSCs regulate H19 transcript levels in myoblasts under hypoxic stress by co-culturing proliferating primary myoblasts (isolated from the same strain of NOD/SCID mice used in *in vivo* functional assessments) with PGI_2_-hMSCs, 3.1-hMSCs+ILO or 3.1-hMSCs. We found a significant transient increase (more than twofold) in H19 transcript levels in myoblasts co-cultured with PGI_2_-hMSCs as compared with those co-cultured with 3.1-hMSCs (*P*<0.01; one-way ANOVA; Fig. 7a). This early increase in H19 lncRNA seen at 24 h was not maintained at 48 h (Fig. 7a). There was no difference in the total viable cells at 24 h; however, at 48 h, the number of viable myoblasts co-cultured with PGI_2_-hMSCs increased significantly as compared with those co-cultured with 3.1-hMSCs (7.67±0.10 × 10^4^ versus 6.47±0.10 × 10^4^ cells, *P*<0.01; one-way ANOVA; Fig. 7b). Co-culturing myoblasts with 3.1-hMSCs+ILO had similar effects on H19 upregulation and cell survival as did co-culturing with PGI_2_-hMSCs (Fig. 7a,b). Co-culture of PGI_2_-hMSCs significantly reduced the number of nonviable myoblasts at both 24 and 48 h (one-way ANOVA; Supplementary Fig. 3a). However, the total number of nonviable cells under all treatment conditions was 10-fold less than the number of viable cells (Fig. 7b; Supplementary Fig. 3a). Therefore, the increase in viable myoblasts at 48 h, after co-culture with PGI_2_-hMSCs or 3.1-hMSCs+ILO, is likely due mainly to increased cell proliferation, with a minor component due to cell survival.

To examine whether increased H19 expression is associated with cell survival and proliferation under hypoxia, we used short interfering RNA (siRNA) to knockdown *H19* in proliferating myoblasts. Downregulation of H19 lncRNA (40%, *P*<0.05; two-tailed *t*-test; Supplementary Fig. 3b) significantly reduced viable myoblasts (4.89±0.16 × 10^4^ [negative control siRNA] versus 3.97±0.09 × 10^4^ [H19 siRNA-transfected myoblasts]) and significantly increased nonviable myoblasts (*P*<0.01; two-tailed *t*-test; Supplementary Figs 3c,d). After knocking down H19 in myoblasts, we performed transwell co-culture assays of PGI_2_-hMSCs and myoblasts carrying H19 knockdown (4 × 10^4^ cells) or PGI_2_-hMSCs and myoblasts carrying scrambled siRNA (4 × 10^4^ cells, serving as a control) for 48 h, in parallel, under hypoxia. In comparison with controls, *H19* knockdown led to a significant decrease in the total number of viable myoblasts (*P*<0.01; two-tailed *t*-test; Fig. 7c) and a significant reduction in cell proliferation (*P*<0.01; two-tailed *t*-test; Fig. 7d). This finding indicates that PGI_2_-hMSC-induced cytoprotection of progenitor cells under hypoxia is H19 dependent. Supporting the above results, we found that PGI_2_-hMSCs and 3.1-hMSCs+ILO upregulate H19 lncRNA and exert similar cytoprotective effects on C2C12 myoblasts as compared with primary myoblasts (Supplementary Figs 4a–d). Moreover, H19 RNA levels did not differ significantly among C2C12 cells in the absence or presence of ILO (100 nM; Supplementary Fig. 4e). These findings indicate that both PGI_2_ (or analogues) and additional paracrine factors released from hMSCs are required to stimulate H19 expression in myoblasts under hypoxia.

**Figure 8:**
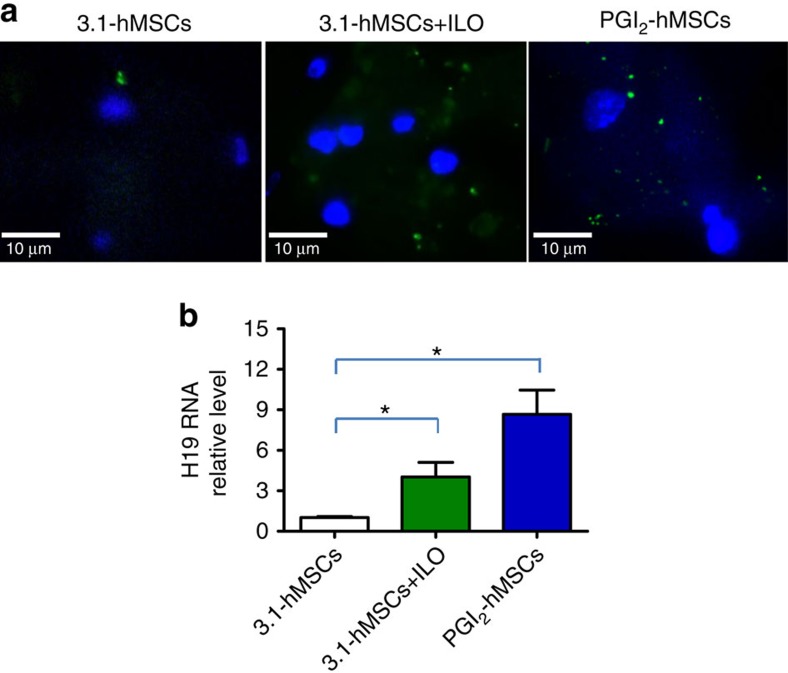
Upregulation of H19 lncRNA in ischaemic hindlimbs treated with PGI_2_-hMSCs. (**a**) Representative images of H19 RNA fluorescence *in situ* hybridization in gastrocnemius muscle sections at 3 days after 3.1-hMSC, 3.1-hMSC+ILO or PG_2_-hMSC injections. We used a fluorescein-tagged H19 FISH probe that specifically targets endogenous H19 lncRNA, resulting in intense intracellular green fluorescent particles. We found a higher expression of host H19 lncRNA in PGI_2_-hMSC- and in 3.1-hMSC+ILO-treated muscles than in 3.1-hMSC-treated muscles (*n*=3 mice per group). All the sections were counterstained with 4,6-diamidino-2-phenylindole (DAPI) to localize nuclei. (**b**) Quantitative RT–PCR showed a significant increase in H19 lncRNA levels in ischaemic gastrocnemius muscle 5 days after injections with 3.1-hMSCs+ILO or PG_2_-hMSCs as compared with tissue samples injected with 3.1-hMSCs. *P<0.05 by one-way ANOVA with Newman–Keuls *post hoc* test. Data are shown as mean±s.e.m. from three independent assays. *N*=3 mice (male and female) per group. Mice were sex matched among the groups. Scale bar, 10 μm.

After confirming that PGI_2_-hMSCs trigger H19 lncRNA upregulation in myoblasts *in vitro* during low-oxygen tension, we examined the effect of PGI_2_-hMSCs on endogenous H19 lncRNA expression in ischaemic hindlimbs using RNA fluorescence *in situ* hybridization (RNA-FISH). A mouse H19 fluorescent oligonucleotide probe was used to detect single H19 lncRNA molecules. H19 sequences that contain miR-675-3p and miR-675-5p were excluded in the probe design to avoid non-specific binding^20^. RNA-FISH studies showed an increase in H19 lncRNA levels in the cytoplasm of endogenous cells surrounding PGI2-hMSC injection sites as compared with 3.1-hMSC injection sites at 3 days after cell administration (Fig. 8a). Quantitative PCR with reverse transcription (RT–qPCR) further showed that levels of H19 RNA were significantly higher in gastrocnemius muscle treated with PGI_2_-hMSCs than in that treated with 3.1-hMSCs, suggesting that PGI_2_-hMSCs induce an upregulation of endogenous H19 lncRNA in ischaemic hindlimbs (one-way ANOVA; Fig. 8b).

## Discussion

In the present study, we have shown that hMSCs engineered to produce PGI_2_ improved distal perfusion of the lower extremity, enhanced exercise endurance and increased muscle mass in a mouse hindlimb ischaemia model. The benefits achieved with PGI_2_-hMSCs were superior to those seen with control hMSCs or ILO, indicating that treatment with PGI_2_-hMSCs results in greater tissue healing and repair. Interestingly, functional recovery was not induced by the prolonged presence of PGI_2_-hMSCs but rather by an improved mobilization of the host response. In support of this finding, we found that higher numbers of Sca-1^+^Ki67^+^ resident progenitor cells localized at PGI_2_-hMSCs injection sites than at 3.1-hMSCs injection sites 3 days after cell delivery. Moreover, our *in vitro* data showed that PGI_2_-hMSCs confer pro-survival and pro-proliferative benefits on proliferating myogenic progenitor cells. Mechanistically, we found that the paracrine effects of PGI_2_-hMSCs on progenitor cells were achieved by modulating H19 lncRNA. PGI_2_-hMSCs induced upregulation of H19 lncRNA levels in target cells, which was accompanied by a simultaneous reduction in progenitor cell death. The critical role of *H19* in promoting cell survival/proliferation under hypoxia was confirmed by targeted gene silencing studies. Finally, we present evidence of the *in situ* detection of PGI_2_-hMSC-induced upregulation of H19 lncRNA in mouse ischaemic hindlimbs. To our knowledge, this is the first study to show that genetically modified hMSCs augment the function of resident progenitor cells by regulating lncRNA.

Our data suggest a new mechanism for the paracrine effects of hMSCs—the targeting of lncRNAs. lncRNAs, an array of non-protein coding transcripts over 200 nucleotides long^21^, have emerged as critical transcriptional or post-transcriptional regulators of cellular activity^16^. *H19* is a maternally imprinted gene that is abundantly expressed during embryonic development^22^. After birth, *H19* expression continues in skeletal muscle. Upregulation of H19 lncRNA in myoblasts has been proposed to affect differentiation and myogenesis^18^,^19^; however, the cytoprotective properties of H19 lncRNA on progenitor cells have not been elucidated. Here we found a link between H19 lncRNA upregulation and myogenic progenitor cell survival under hypoxia; enhanced H19 expression protected myoblasts from death. In contrast, targeted H19 lncRNA knockdown led to an increase in nonviable cells and decreased cell proliferation. Our data suggest that H19 lncRNA may act as an early regulatory element in augmenting cellular adjustment to environmental stress. This adaptation might not immediately translate into growth but instead induces mechanisms of protection. As a result, H19 lncRNA imparts cells with increased resistance to stress and provides a growth advantage. Although our study points to a critical role in cytoprotection for H19 lncRNA, its role in promoting cell growth is still under investigation. H19 lncRNA may promote cellular proliferation by modulating downstream target genes, as we detected higher numbers of viable cells but not differential expression of H19 lncRNA at 48 h after PGI_2_-hMSC co-culture (Fig. 7; Supplementary Fig. 3). However, we cannot rule out a direct effect of H19 lncRNA on regulating genes related to promoters or inhibitors of the cell cycle as knockdown of H19 lncRNA decreased cell growth. The current study included only limited time points; a more detailed time-course study of H19 lncRNA expression and its biological relevance is warranted.

The creation of hMSCs that secrete PGI_2_ overcomes a major hurdle of current PGI_2_ therapy. As a vasodilatory drug, PGI_2_ has multiple favourable properties for treating ischaemia, including mediating vascular homeostasis, and inhibiting thrombosis and platelet aggregation^23^,^24^,^25^. Although PGI_2_ therapy can be used to avoid open surgical revascularization in high-risk patients such as the elderly^26^, it has a major disadvantage. PGI_2_ is an unstable compound with a circulating half-life of 1–2 min and must be administered using a continuous pump with an indwelling catheter^27^. This cumbersome delivery system greatly reduces the patient's quality of life and is associated with significant adverse events. Infection at the infusion site can lead to life-threatening complications. Furthermore, continuous infusion of PGI_2_ is a financial burden. Therefore, a new approach to effectively deliver PGI_2_ is urgently needed for treating patients with ischaemic diseases. Here we utilized hMSCs as a biological vehicle to consistently release PGI_2_ directly into ischaemic tissues. This not only helps prevent adverse events caused by current PGI_2_ delivery methods but also offers a novel cellular therapeutic approach that can increase PGI_2_ biosynthesis in ischaemic areas.

Our results show that PGI_2_-hMSCs provided benefits that were superior to those seen with native hMSCs or ILO alone. These findings establish a paradigm for using a promising cell candidate to produce a clinically relevant reagent that may enhance the therapeutic potential of native cells or genes. In the current study, we used PGI_2_ as an agent for controlling cell function because PGI_2_ exerts broad favourable effects on endogenous stem cells. He *et al*. found that secretion of PGI_2_ by human outgrowth EPCs facilitates vascular regeneration^6^. In contrast, inhibiting PGI_2_ production in EPCs reduced cell proliferation, survival and angiogenic capacity in mouse ischaemic hindlimbs^6^. Moreover, PGI_2_ signalling promotes the migration and recruitment of EPCs to injured vessels^28^. Together, these studies highlight the capacity of PGI_2_ to enhance the natural abilities of stem cells.

Treadmill exercise performance is a useful clinical test for evaluating the functional outcome of pharmacological therapy. To understand how PGI_2_-hMSC therapy improved exercise capacity in mice with acute limb ischaemia, we measured the mass of the gastrocnemius muscle in ischaemic hindlimbs 1 month after cell or drug treatment. Our results suggested possible underlying mechanisms that led to enhanced running after cell, ILO or combination therapy. First, we found that mice receiving daily ILO for 21 days did not show increased muscle mass but did show increased running distance over mice treated with vehicle or 3.1-hMSCs. This enhanced performance may be due to improved systemic functions rather than to muscle build-up. PGI_2_ and its stable analogues such as ILO are potent vasodilators. Systemic administration (for example, intraperitoneal injections) can augment heart and lung tolerance during exercise by relaxing blood vessels, leading to better endurance. However, the sudden termination of long-term drug administration will decrease the stimulant effect on the heart and lungs. This may explain why exercise performance declined by 30% 1 week after ILO was discontinued. Second, we found that muscle build-up may have contributed to enhanced exercise performance in mice treated with 3.1-hMSCs+ILO. This finding implies the synergistic effects of ILO and cell therapy in promoting muscle gain. While the importance of muscle mass build-up on performance may be initially under-appreciated as exercise capability was similar between mice receiving 3.1-hMSCs+ILO and ILO treatment alone, its role became more apparent after ILO was discontinued. One week after terminating daily ILO treatment, the relative efficacy of the 3.1-hMSCs+ILO group compared more favourably to the 3.1-hMSC group than did the ILO group. Mice in the 3.1-hMSCs+ILO combination group ran a significantly longer distance than those receiving 3.1-hMSC treatment, whereas no significant difference was found between those treated with ILO and 3.1-hMSCs. Finally, we found that a single dose of PGI_2_-hMSCs into the ischaemic hindlimbs promoted a similar level of muscle mass gain as did treatment with 3.1-hMSCs+ILO. Given that muscle gain requires concurrent angiogenesis and myogenesis, it is likely that PGI_2_ and other paracrine factors released from PGI_2_-hMSCs acted synergistically to facilitate cell survival/growth and to increase capillary formation for nourishing regenerating fibres in a hostile microenvironment. Although PGI_2_-hMSC therapy yields multiple beneficial effects, the treadmill test also indicated that mice vary in their response to treatment regimens.

The dynamic interplay of multiple signalling pathways during the regenerative process may be one of the critical mechanistic explanations of the positive effects of cell therapy. Secretome characterization at any one time point may be helpful in identifying the signalling pathways that contribute to clinical benefits; however, this approach may not accurately reflect the consistent subtle shift in the secretome (for example, timing of release, duration, levels relative to other simultaneously released elements) that results from changes in the microenvironment. Thus, we developed a novel strategy that uses PGI_2_ as a master regulator to enhance the overall paracrine effects of hMSCs on the endogenous regenerative responses in ischaemic tissues. We used PGI_2_-hMSCs as a biological tool to endow proliferating endogenous cells with more resistance to ischaemic injury. The positive outcome of our approach has been confirmed by both *in vivo* and *in vitro* studies as presented here.

## Methods

### Transfection of human mesenchymal stem cells

Human bone marrow-derived mesenchymal stem cells (hMSCs, between passages 3 and 4; Lonza (PT2501), Switzerland) were transfected by electroporation (Human MSC Nucleofector Kit, Lonza) to introduce a plasmid pcDNA 3.1 (Invitrogen, Carlsbad, CA, USA) or a pcDNA 3.1 expressing a triple-catalytic hybrid enzyme that links COX-1 to PGIS (the hybrid enzyme [COX-1-10aa-PGIS])^8^. After nucleofection, cells were grown under G418 (200 μg ml^−1^) selection, and confluent cell monolayers were collected for evaluation of stable expression of the transgene COX-1-10aa-PGIS. hMSCs containing pcDNA 3.1 were referred to as 3.1-hMSCs and those containing pCOX-1-10aa-PGIS were referred to as PGI_2_-hMSCs.

### PCR and western blot and enzyme immunoassays of hMSCs

Genomic DNA was isolated and purified from native hMSCs, 3.1-hMSCs and PGI_2_-hMSCs according to the manufacturer's protocol (DNeasy Blood and Tissue Kit, Qiagen, Germantown, MD, USA). PCR was performed using total DNA (200 ng per sample), COX-1-10aa-PGIS-specific primers (forward primer sequence: 5′-CCTCAAGGGTCTCCTAGGGTA-3′; reverse primer sequence: 5′-GTGCTTCTCCTTCATCCTCGT-3′), and platinum Taq DNA polymerase (Invitrogen).

The size of PCR product (445 bp) that spans the 10-aa linker region of COX-1-10aa-PGIS was determined by comparison with a 100-bp DNA ladder under ultraviolet light after running 2% agarose gel electrophoresis^16^. Cell lysates prepared from hMSCs, 3.1-hMSCs and PGI_2_-hMSCs were used to assess the expression of fusion protein (COX-1-10aa-PGIS) by western blot. In brief, 5 μg of protein was fractionated by polyacrylamide gel electrophoresis (SDS–PAGE; 4–20% gradient gel, Bio-Rad, Hercules, CA, USA) and transferred onto a polyvinylidene fluoride membrane. The membrane was incubated with a primary antibody against human COX-1 (1:1,000 dilution, Cayman Chemical, Ann Arbor, MI, USA) followed by an horseradish peroxidase (HRP)-conjugated anti-mouse secondary antibody (1:5,000 dilution, Sigma, St Louis, MO, USA). Protein signals were detected using the ECL system (Thermo Scientific, Rockford, IL, USA). To verify equal loading of each protein sample, we stripped the membranes and re-probed them with β-actin monoclonal antibody (1:1,000 dilution, Sigma). All uncropped western blots can be found in the Supplementary Information.

The secretions of PGI_2_ in the supernatants from native hMSCs, 3.1-hMSCs and PGI_2_-hMSCs were measured using the 6-keto prostaglandin F1α enzyme immunoassay (6-keto prostaglandin F1α EIA kit, Cayman Chemical) according to the manufacturer's instructions. Briefly, the supernatant was collected after incubating cells (4 × 10^4^) with arachidonic acid (20 μm in mesenchymal stem cell basal medium) for 20 min at 37 °C. The absorbance was read using a microplate reader (Safire II, Tecan, Triangle Park, NC, USA), and the concentration (pg ml^−1^) of 6-keto prostaglandin F1α was calculated for each sample using formulas provided by Cayman Chemical Company.

### Lentiviral package and transduction of hMSCs

We maintained 293-METR and 293-MSR cells in complete media consisting of DMEM supplemented with HEPES, G418 (Invitrogen), Puromycin, Normocin, Zeocin (Invivogen) and 10% heat-inactivated fetal bovine serum (GE Healthcare Life Science Hyclone Laboratories, South Logan, Utah, USA). To package lentiviruses, we first seeded 1.5 × 10^7^ 293-METR cells in a 150 × 25-mm dish with 20 ml of DMEM 20 h before transfection, and then we added the DNA/FuGENE complex to the cells for transfection. The complex was made with the plasmid DNA mixture, which consisted of 30 μg of lentiviral vector pLV450G nesTML (Supplementary Fig. 2a), 19.6 μg of psPAX2 and 12.6 μg of pMD2.G in 3.2 ml of DMEM incubated for 25 min at room temperature, and 192 μl of FuGENE HD Reagent (Promega, Madison, WI, USA). To collect the packaged lentivirus, we collected the DMEM ∼28–30 h after transfection, replaced it with 20 ml fresh DMEM and recollected the DMEM 24 h later. All samples of virus-containing DMEM were stored at 4 °C, pooled and concentrated by PEG-*it* Precipitation (System Biosciences, Mountain View, CA, USA). The concentrated lentivirus was titred by limiting dilution on 293-MSR.

hMSCs were cultured in supplemented MSCGM medium according to the manufacturer's specifications (Lonza). We transduced hMSCs (at passages 3–4) with the concentrated lentivirus at a multiplicity of infection of 30. The transduction efficiency was assessed by measuring mCherry expression in the lentivirus-transduced hMSCs (LV-hMSCs) via fluorescent microscopy (Supplementary Fig. 2b) and flow cytometry (FACS, BLR-II, BD Biosciences, CA, USA; Supplementary Fig. 2c).

### Mouse unilateral hindlimb ischaemia model

All animal procedures were conducted according to the University of Texas Health Science Center Animal Welfare Committee guidelines in accordance with the National Institutes of Health Guide for the Care and Use of Laboratory Animals. We randomly divided NOD/SCID mice (NOD/ShiLtSz-*Prkdc*^*scid*^/J mice of both sexes, 11–12 weeks old; Jackson Laboratory, Bar Harbor, ME, USA) into five treatment groups: vehicle (PBS), 3.1-hMSCs, ILO (Cayman Chemical), 3.1-hMSCs+ILO or PGI_2_-hMSCs. To create unilateral hindlimb ischaemia, we surgically ligated the left femoral artery in mice anaesthetized by isoflurane inhalation (2–4% isoflurane in oxygen). Specifically, we placed two adjacent sutures on the femoral artery, proximal to the origin of the femoral bifurcation, to interrupt flow^29^. The incision was closed, and the mice were returned to their cages. At 24 h after surgery, we injected PBS, 3.1-hMSCs (4.5 × 10^5^) or PGI_2_-hMSCs (4.5 × 10^5^) into the gastrocnemius muscle of the ischaemic hindlimbs. For the ILO or 3.1-hMSCs+ILO groups, beginning at 24 h after surgery, we administered an intraperitoneal injection (2 ng kg^−1^ per day) of the drug once daily for 21 days; the daily injection was performed after various measurements to avoid the immediate effects of ILO on hemodynamics such as mean arterial pressures. To measure muscle mass after treatment, we killed the mice by CO_2_ inhalation at day 30 and then measured body weight and mass of the gastrocnemius muscle of the ischaemic hindlimb (*n*=9 mice/PBS; *n*=5/ILO; *n*=10/3.1-hMSCs; *n*=10/3.1-hMSCs+ILO; *n*=10/PGI_2_-hMSCs).

### Laser Doppler perfusion imaging

We made serial measurements of perfusion with the use of a laser Doppler image device (Perimed AB, Germany) at 24 h, and at 3, 5, 7 and 14 days after cell injections in the cell-treated groups (*n*=10 per group) and at the same time points in vehicle (*n*=16) and ILO-treated mice (*n*=10)^30^. Perfusion was expressed as the perfusion ratio in the ischaemic compared with the contralateral, non-manipulated leg.

### Running endurance

At 21 and 28 days, mice in the various treatment groups were challenged with acute exercise in a run-to-exhaustion study. Before running, mice were acclimated to the treadmill (Eco 3/6, Columbus Instruments, Columbus, OH, USA; inclination +5°) for 1–2 h and to the motor sound for 15 min. At the start, the belt was set at a slow speed (6 m min^−1^), and the treadmill velocity was increased 2 m every 2 min for the initial 12 min and held constant (18 m min^−1^) thereafter. Exhaustion was defined as the point when mice spent >10 consecutive seconds on the shock grid without trying to reengage the treadmill. We recorded the running time and distances.

### Bioluminescence imaging

We used the Xenogen IVIS 200 system (Xenogen, Alameda, CA, USA) to quantitatively track LV-hMSCs. To establish the correlation between the photon flux of the bioluminescence imaging (BLI) signal and the numbers of LV-hMSCs, we plated serially diluted (2.5 × 10^4^ to 4.0 × 10^5^) LV-hMSCs into six-well plates and added 1 μl of D-luciferin (40 mg ml^−1^ stock; Xenogen) into 2 ml of culture medium per well. After 30 s, we placed the plates into the Xenogen IVIS 200 system for BLI signal detection (Supplementary Fig. 2d). The BLI signals were quantified in units of maximum photons s^−1^ cm^−2^ sr^−1^. A robust linear correlation was observed between the numbers of LV-hMSCs and the photon flux (Supplementary Fig. 2e).

To track the injected LV-hMSCs, mice were intraperitoneally injected with D-luciferin (150 mg kg^−1^) and imaged using the similar Xenogen IVIS 200 system for 15 s at 2-min intervals until maximum photon levels were reached. The mice were scanned at 24 h, and at 3, 5, 7 and 14 days after cell injections in the cell-treated groups (*n*=5 per group) and at the same time points in PBS-treated mice (*n*=5). The cutoff threshold value of *in vivo* BLI signal was set at 1 × 10^6^ photons s^−1^ cm^−2^ sr^−1^ in Fig. 2a.

### Genomic PCR of tissue samples

Mice (*n*=2 per group) were euthanized by CO_2_ inhalation at 14 days after cell, drug or vehicle delivery. Heart, spleen, lung, liver, kidney and muscle were collected, and the individual tissue or organ mass was measured. Genomic DNA was isolated using QIAamp DNA Mini Kit (Qiagen) according to the manufacturer's instructions. DNA concentration and quality were determined by nanodrop 1000 (Thermo Scientific). PCR was performed using total DNA (200 ng per sample) and DNA platinum Taq DNA polymerase (Invitrogen). Primers were designed previously and were used to selectively detect the human *HLA-A* gene (forward primer sequence: 5′-GCTCAGTTCCAGTTGCTTG-3′; reverse primer sequence: 5′-GCAGTGAGCCAAGATTGCAC-3′)^9^. The size of PCR products (214 bp) was determined by comparison with a 100-bp DNA ladder (Invitrogen) using multiImage II with AlphaView (Alpha Innotech, Miami, FL, USA) after 2% agarose gel electrophoresis. The positive control contained a mixture of 20 ng of DNA from hMSCs and 100 ng of DNA from the gastrocnemius muscle of vehicle-treated mice. The negative control contained 100 ng of DNA from the gastrocnemius muscle of vehicle-treated mice.

### Immunofluorescence and haematoxylin and eosin staining

Mice were euthanized by CO_2_ inhalation at 3 days (*n*=3 mice per group) at the end of the treatment regimen, and the gastrocnemius muscle of the ischaemic hindlimb was excised. The muscle sections were fixed in 4% paraformaldehyde (USB Corporation, Cleveland, OH, USA) at 4 °C overnight, and then incubated in 10–30% (w/v) sucrose solution at 4 °C overnight. The sections were embedded in Tissue-Tek OCT compound (VWR, Radnor, PA, USA) and stored at −80 °C freezer^5^. We incubated the cross-sections of muscle tissue (6 μm) overnight at 4 °C with the following primary antibodies individually or in combination: anti–Ki67 (1:100 dilution; Abcam, Cambridge, MA, USA) and anti-Sca-1 (1:50 dilution; Biolegend, San Diego, CA, USA). The sections were then incubated with corresponding secondary antibodies (1:250 dilution): Alexa Fluor-647 donkey anti-rabbit IgG (for Ki67 detection, pseudo-white in Fig. 5), Alexa Fluor-488 goat anti-rabbit IgG (for Ki67 detection in Fig. 4) or Alexa Fluor-488 donkey anti-rat IgG (all from Invitrogen). Nuclei were counterstained with 4,6-diamidino-2-phenylindole. We used a confocal laser scanning microscope (Leica TCS SP5II, Buffalo Grove, IL, USA) to obtain fluorescence images of stained sections. Image processing and quantitative analysis were performed using the ImageJ software (http://imagej.nih.gov/ij/). To quantify Ki67^+^Sca-1^+^ and Ki67^+^Sca-1^−^ cells, we analysed a total of 30 high-power fields (HPFs) from three mice per treatment group (10 HPFs per mouse). The HPF was chosen to have approximately similar numbers of cells as a whole based on the enumerated nuclei, including five HPFs at the centre of the injection site, three HPFs from the proximal region and two from the distal region per gastrocnemius muscle. For haematoxylin and eosin staining, mice were euthanized at 14 days after treatment (*n*=2 mice per group). The gastrocnemius muscle of the ischaemic hindlimb was excised and embedded in paraffin blocks. Tissue sections (6 μm) were deparaffinized, rehydrated and stained with eosin (Sigma), and then counterstained with haematoxylin (Sigma). We used an Olympus microscope (BX-51) to obtain the images.

### Human proteome profiler antibody array

We seeded 3.1-hMSCs, 3.1-hMSCs+ILO (3.1-hMSCs were pretreated with 100 nm of ILO for 7 days) and PGI_2_-hMSCs (5.0 × 10^5^ per treatment) onto culture dishes with MSC growth medium (MSCGM, Lonza). The cultures were placed in a 5% CO_2_ incubator at 37 °C for 24 h, and then washed and incubated with MSCGM (with 0.1% BSA) in the absence of mesenchymal cell growth supplement in a hypoxic incubator (1.5% O_2_, New Brunswick Galaxy 14 S, Eppendorf, Enfield, CT, USA) for 72 h. The culture media were collected and centrifuged at 1,000 r.p.m. for 10 min to remove debris. The soluble proteins in media were concentrated, by a factor of 25 × at 4 °C, using Amicon Ultra-15 centrifugal filter units (3 kDa cutoff, EMD Millipore, Billerica, MA, USA) before use in the antibody array. The human angiogenesis array (ARY007, R&D systems, Minneapolis, MN, USA) was performed according to the manufacturer's instructions. Quantitative analyses were performed using the ImageJ software (http://imagej.nih.gov/ij/).

### Human MSC and myoblast co-culture

After euthanizing NOD/SCID mice (11–12 weeks old; both sexes), we exposed the limb muscles, dissected away the bones and fat, and minced the muscle tissue to a slurry with surgical scissors. The muscle slurry was incubated with collagenase type II (0.1 mg ml^−1^, Worthington Biochemical Corp, Lakewood, NJ, USA) at 37 °C for 20 min on a shaker, followed by low-speed centrifugation (500 r.p.m.). We removed the supernatant, resuspended the pellet with collagenase/dispase (1 mg ml^−1^, Roche Applied Science, Indianapolis, IN, USA) and incubated it at 37 °C for 30 min. During the incubation period, the slurry was gently triturated with pipetting every 8–10 min. The homogenate was filtered sequentially with 100-, 70- and 40-μm cell strainers, and was centrifuged at 1,200 r.p.m. for 10 min. The supernatant was removed, and the pellet was resuspended in DMEM growth medium and plated in 100-mm Petri dishes in a 5% CO_2_ incubator at 37 °C for 1 h. Then, the supernatant containing enriched myoblasts was transferred and grown in collagen-coated culture dishes in complete DMEM growth medium^31^,^32^. Primary myoblasts (4 × 10^4^ cells) or mouse C2C12 myoblasts (5 × 10^4^ cells, ATCC, Manassas, VA, USA) were seeded in tissue culture plates and cultured with growth medium (DMEM medium [ATCC] containing 10% fetal bovine serum [ATCC] and 1% penicillin-streptomycin [Lonza]) in a 5% CO_2_ incubator at 37 °C for 24 h. We placed transwell inserts (0.4-μm pore size; BD Biosciences; San Jose, CA, USA) containing 3.1-hMSCs, 3.1-hMSCs+ILO (100 nm) or PGI_2_-hMSCs (5 × 10^4^ cells per well in C2C12 co-cultures or 4 × 10^4^ cells per well in primary myoblast co-cultures) into each well. The cells were co-cultured in growth medium in a hypoxic incubator (1.5% O_2_) for 24 or 48 h. In parallel, C2C12 cells were grown in the absence of any treatment or in the presence of ILO (100 nm). We removed the inserts and processed the myoblasts for viability assays, RT–qPCR or other analyses.

### Cell viability assay

We used the trypan blue exclusion assay to obtain counts of viable and nonviable myoblasts. The assay was performed according to the online protocol provided by Life Technologies (http://www.lifetechnologies.com/us/en/home/references/gibco-cell-culture-basics/cell-culture-protocols/trypan-blue-exclusion.html). We performed all experiments in quadruplicate in three independent experiments.

### RT–qPCR

After the treatments described above, myoblasts were washed with cold Dulbecco's phosphate-buffered saline (DPBS) and collected for RNA isolation (RNase Plus Micro Kit, Qiagen). To prepare tissue for RNA isolation, we micro-dissected cell injection areas of the gastrocnemius muscle of ischaemic legs after 5 days (*n*=3 per group). The snap-frozen samples were homogenized by mortar and pestle and placed in a bioruptor-sonicator (Diagenode, Denville, NJ, USA). Total RNA (2 μg) was reverse transcribed using high-capacity RNA-to-cDNA kit (Invitrogen) and T100 thermal cycler (Bio-Rad, Hercules, CA, USA). qPCR was performed using TaqMan Gene Expression Master Mix (Invitrogen) and 7900HT Fast Real-Time PCR System (Life Technologies, Grand Island, NY, USA). *H19*-specific primers/probes and 18S rRNA endogenous control (VIC/MGB Probe) were purchased from Life Technologies. The relative expression of RNA was calculated using RQ Manager 1.2.1(the ΔΔCt method). All experiments were performed in triplicate in three independent experiments.

### H19 knockdown

RNAiMAX transfection reagent, silencer select H19 siRNAs (n253569 and n253570), a negative control set (scrambled siRNA) and opti-MEM medium were purchased from Invitrogen. H19 siRNA was transfected into myoblasts according to the manufacturer's procedures. Negative control siRNA was transfected in parallel. Mouse C2C12 myoblasts (5 × 10^4^ per well) or primary myoblasts (4 × 10^4^ cells) were seeded and cultured with growth medium in a 5% CO_2_ incubator at 37 °C for 24 h (day 1) before transfection. We transfected H19 siRNA (n253569; 100 pmol) into myoblasts at day 2, and then H19 siRNAs (n253569 and n253570; 100 pmol) at day 3 to ensure sufficient knockdown. Six hours after the second transfection, the cells were transferred to a hypoxic incubator (1.5% O_2_) for an additional 42 h before being collected for various analyses.

After confirming H19 knockdown in myoblasts, we co-cultured PGI_2_-hMSCs (4 × 10^4^ cells per well in transwell inserts) with primary myoblasts (4 × 10^4^ cells, treated with scrambled siRNA) or PGI_2_-hMSCs (4 × 10^4^ cells per well in transwell inserts) with primary myoblasts carrying H19 knockdown (4 × 10^4^ cells) in growth medium in a hypoxic incubator (1.5% O_2_) for 48 h. Trypan blue exclusion assay was used to count viable myoblasts. Myoblast proliferation was quantified using cell proliferation ELISA according to the manufacturer's instructions (Roche Applied Science, Indianapolis, IN, USA). We measured 5-bromo-2′-deoxyuridine incorporation into cellular DNA on a microplate reader (A370–A492 nm, Safire II, Tecan). All experiments were performed in quadruplicate in three independent experiments.

### H19 lncRNA RNA-FISH

Mice were euthanized by CO_2_ inhalation at 3 days (*n*=3 mice per group) after cell delivery. Cryosections of the gastrocnemius muscle from cell-treated ischaemic hindlimbs were used for RNA-FISH according to the protocol provided by Biosearch Technologies Inc (Petaluma, CA, USA). An H19 probe labelled with fluorescein was designed against mouse H19 transcript using the online Stellaris FISH Probe Designer version 4.0. The nucleotide sequences containing miR-675-3p and miR-675-5p were excluded from the design. We used an Olympus BX-51 microscope with an oil immersion lens (100 × 1.4 numerical aperture) and an Olympus DP70 digital camera to obtain the images. The light source was an X-cite 120PC Mercury lamp (EXFO).

### Statistical analysis

The data were expressed as the mean±s.e.m. To determine statistical significance among more than two treatment groups, we used a one-way ANOVA following either with Dunnett's multiple comparison test or with Newman–Keuls multiple comparison test (Newman–Keuls *post hoc* test) for pairwise comparison (Graph Pad Prism 5). To determine statistical significance between two groups, we used a two-tailed *t*-test (Graph Pad Prism 5). *P*<0.05 was considered statistically significant.

## Additional information

**How to cite this article:** Deng, Y. *et al*. Prostacyclin-producing human mesenchymal cells target H19 lncRNA to augment endogenous progenitor function in hindlimb ischaemia. *Nat. Commun.* 7:11276 doi: 10.1038/ncomms11276 (2016).

## Supplementary Material

Supplementary Information Supplementary Figures 1-5
