# Supplementary material for: Prostacyclin-producing human mesenchymal cells target H19 lncRNA to augment endogenous progenitor function in hindlimb ischaemia
Source: Nat Commun. 2016 Apr 15;7:11276. doi: 10.1038/ncomms11276 (PMC4835554; doi:10.1038/ncomms11276)
Supplement: Supplementary Information — Supplementary Figures 1-5 [file ncomms11276-s1.pdf]

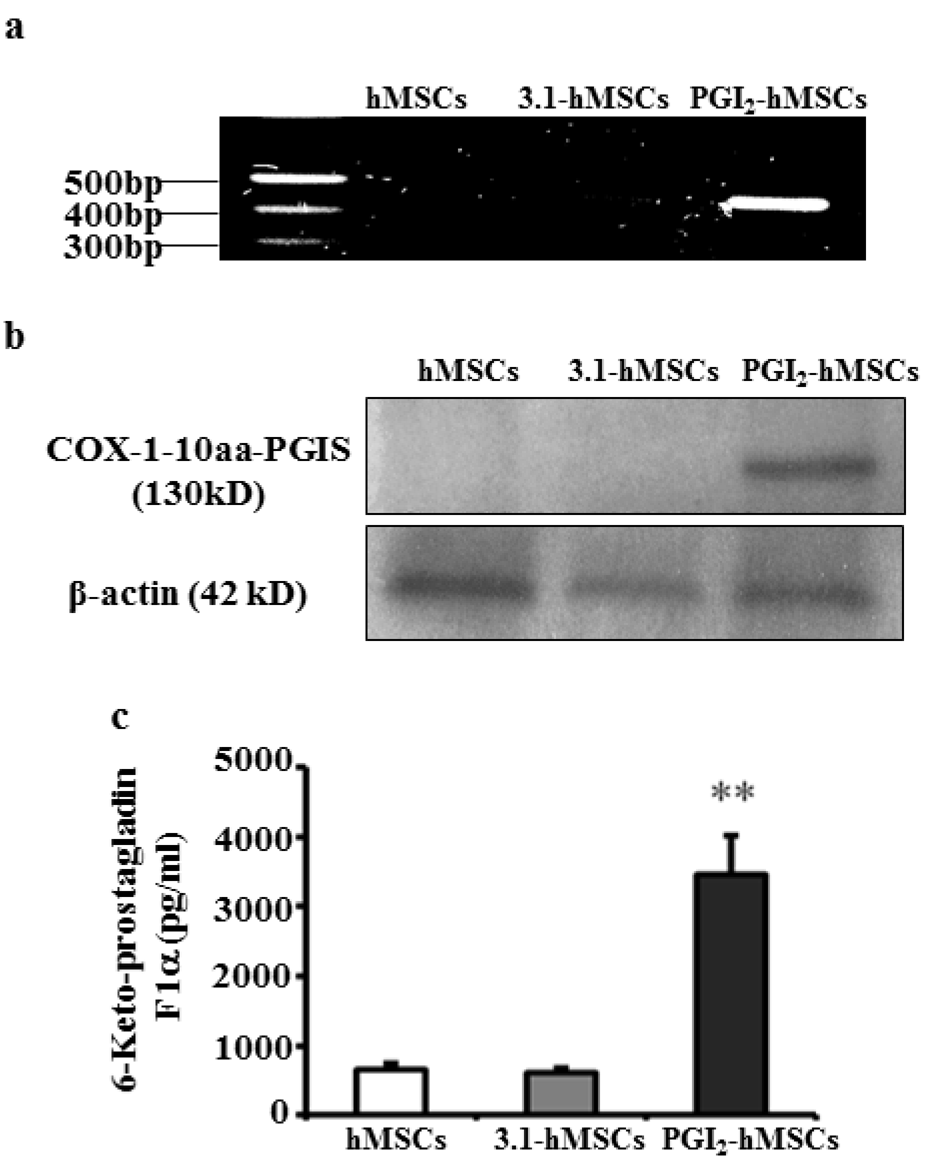

Supplementary Figure 1. Establishment of prostacyclin-secreting hMSCs. (**a**) PCR showed the integration of the COX-1-10aa-PGIS transgene into the genomic DNA of hMSCs (PGI_2-hMSCs). Native hMSCs and plasmid pcDNA 3.1 transfected hMSCs (3.1-hMSCs) were used as negative controls. (**b**) Western blots showed the overexpression of COX-1-10aa-PGIS fusion protein in PGI_2-hMSCs. β-actin was used as a loading control. (**c**) PGI_2-hMSCs produced significantly higher levels of 6-keto PGF1α than did negative controls. $^{**}P<0.01$, PGI_2-hMSCs vs native hMSCs and 3.1-hMSCs. Data are shown as mean±s.e.m from 5 replicates and are representative of 3 independent experiments with similar results. A 1-way ANOVA with Newman–Keuls *post hoc* test was used to determine statistical significance.

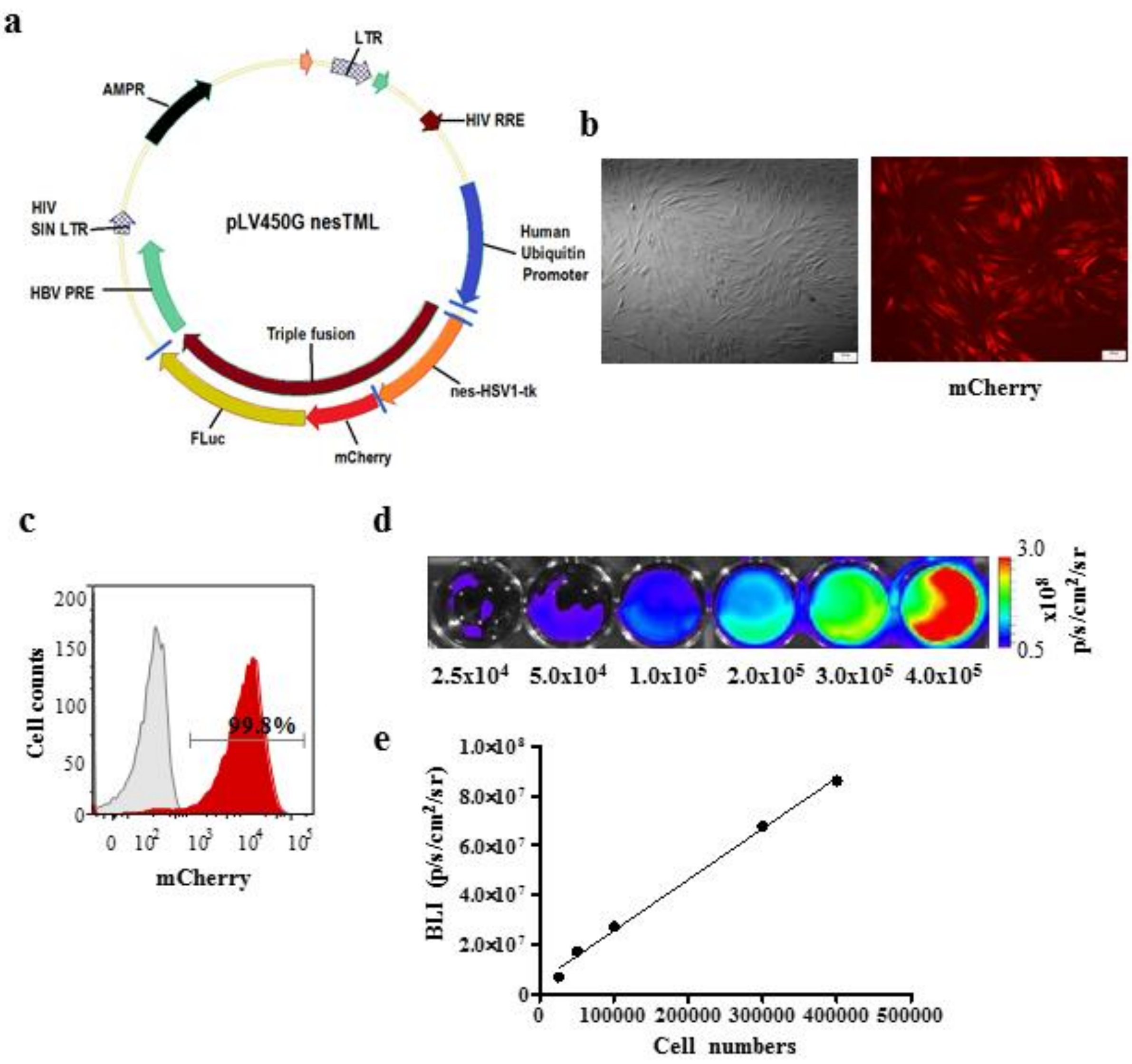

Supplementary Figure 2. hMSCs were efficiently transduced with the lentiviral vector containing triple fusion reporters. (a) Diagrammatic representation of the lentiviral vector encoding genes for herpes virus 1thymidine kinase (HSV1-tk), mCherry fluorophore, and firefly luciferase genes. **(b)** Representative photomicrograph and its corresponding florescence image showing the expression of red florescent mcherry protein in lentiviral transduced hMSCs. Scale bar, 100 μm. **(c)** High efficiency of lentiviral transduction in hMSC was confirmed by flow cytometry analysis. **(d)** Representative in vitro bioluminescent images from hMSCs transduced with the lentiviral vector. Cells were consecutively diluted in a 6-well plate. **(e)** A linear in vitro relationship between bioluminescent signal intensity and cell numbers.

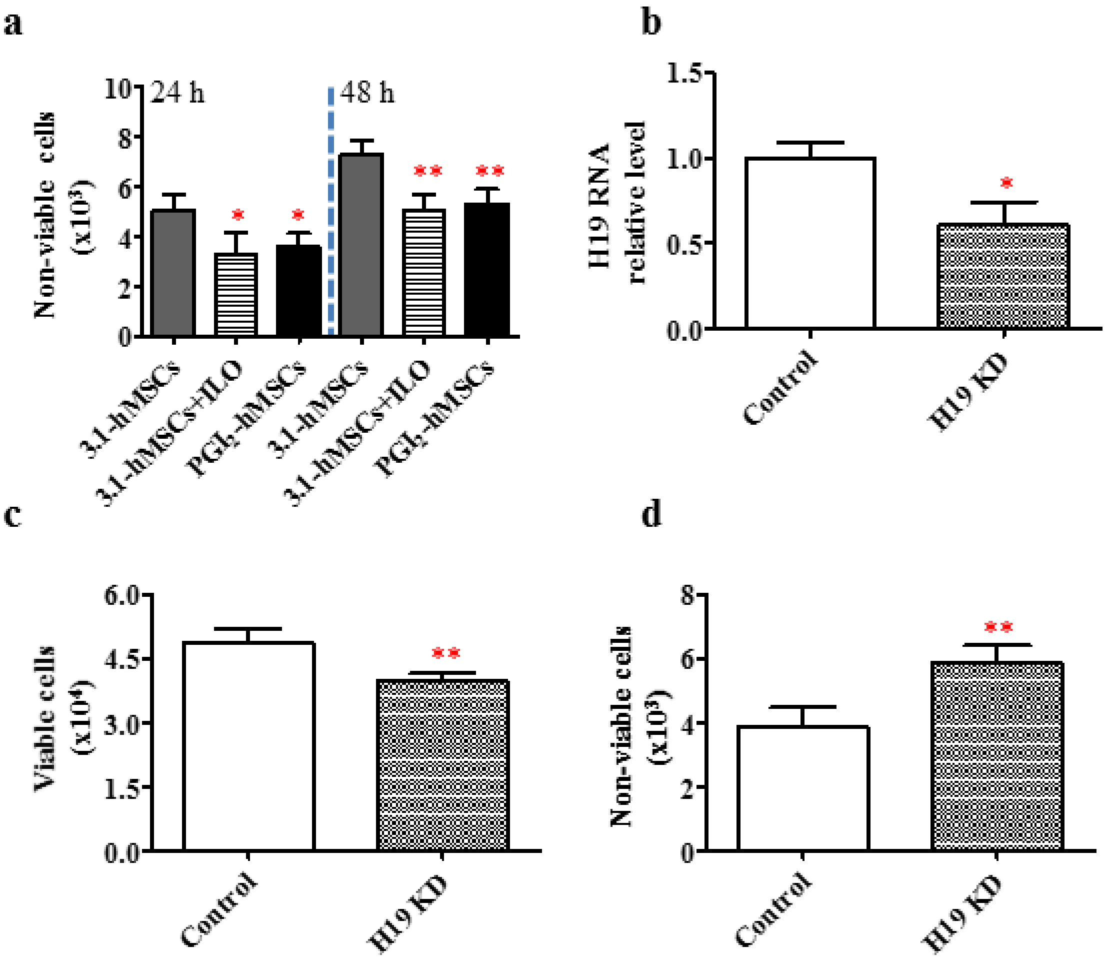

Supplementary Figure 3. Coculturing myoblasts with PGI_2-hMSCs reduced cell death under hypoxic conditions. (**a**) Cell death at 24 and 48 hours was significantly reduced in primary myoblasts that were cocultured with PGI_2-hMSCs or 3.1-hMSCs+ILO as compared with those cocultured with 3.1-hMSCs. (**b**) *H19* silencing by siRNA (H19 KD) significantly reduced H19 levels in primary myoblasts as compared to control myoblasts (transfected with scrambled siRNA). (**c**) *H19* silencing also significantly decreased the number of viable cells and (**d**) increased cell death. *P<0.05; **P<0.01. Statistical significance was determined by 1-way ANOVA with Newman–Keuls *post hoc* test (a) and a two-tailed t test (b,c,d). Data are shown as mean±s.e.m from 3-4 replicates and are representative of 3 independent experiments with similar results.

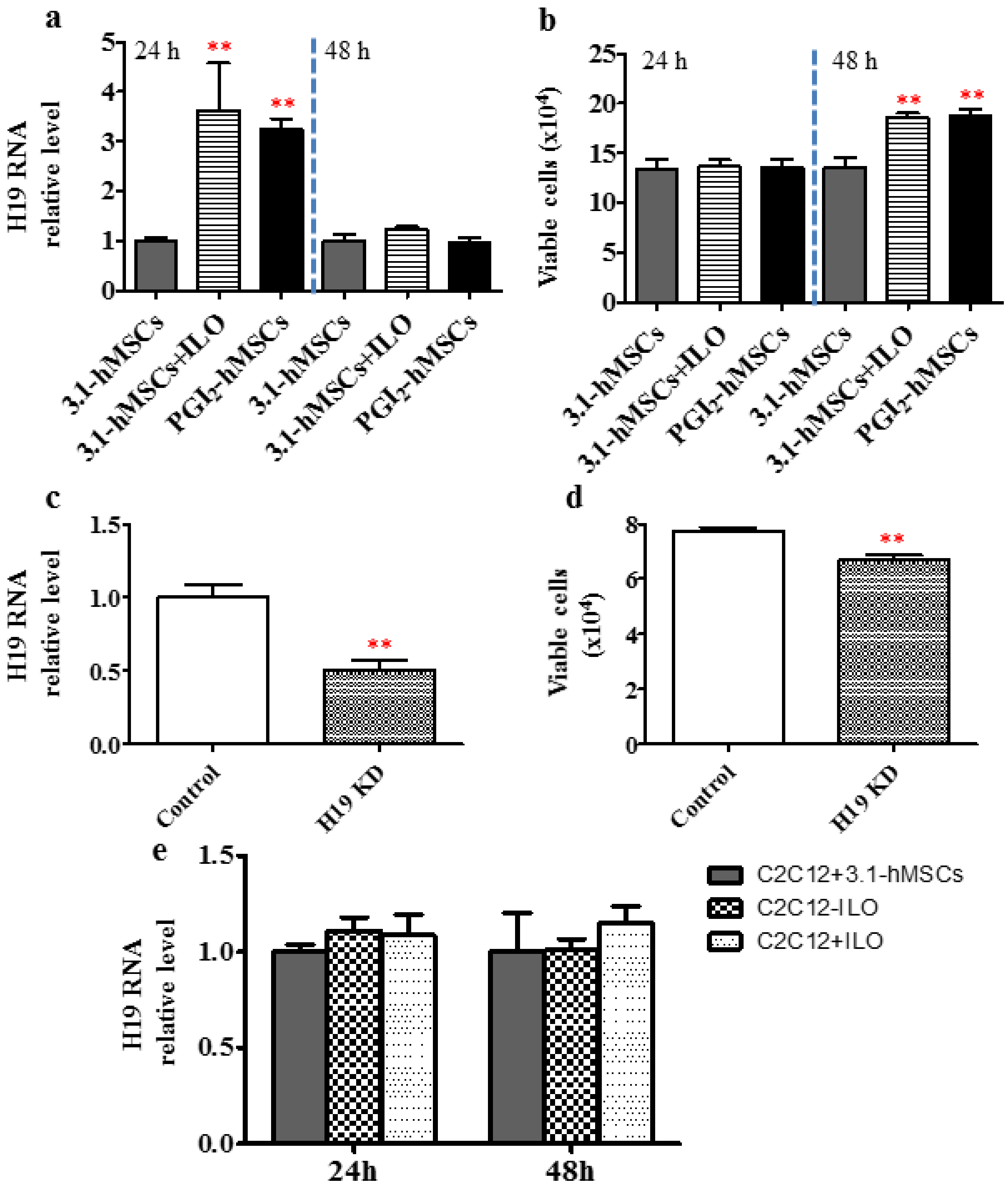

Supplementary Figure 4. H19 lncRNA promoted C2C12 myoblast survival under hypoxia (1.5% O_2). (**a**) H19 transcripts were significantly increased in C2C12 myoblasts after coculture with PG_2-hMSCs or 3.1-hMSCs+ILO in a hypoxic incubator for 24 hours but not for 48 hours as compared to those cocultured with 3.1-hMSCs. (**b**) At 24 hours, the number of viable C2C12

myoblasts was comparable among the 3 cocultured groups, but the number of viable cells increased significantly at 48 hours after coculture with PGI_2-hMSCs or 3.1-hMSCs+ILO. (**c**) H19 lncRNA levels were significantly reduced in C2C12 myoblasts after specific knockdown with H19 siRNA (H19 KD) when compared with negative control siRNA (scrambled siRNA). (**d**) *H19* silencing significantly reduced the number of viable cells. (**e**) The prostacyclin analogue iloprost (ILO) did not stimulate H19 expression in myoblasts under hypoxia. Levels of H19 transcripts were comparable in C2C12 cells not treated with ILO, C2C12 cells treated with ILO, and C2C12 cells cocultured with 3.1-hMSCs in a hypoxic incubator for 24 and 48 hours. $^{**}P<0.01$. Statistical significance was determined by 1-way ANOVA with Newman–Keuls *post hoc* test (a,b) and a two-tailed t test (c,d). Data are shown as mean±s.e.m from 3-4 replicates and are representative of 3 independent experiments with similar results.

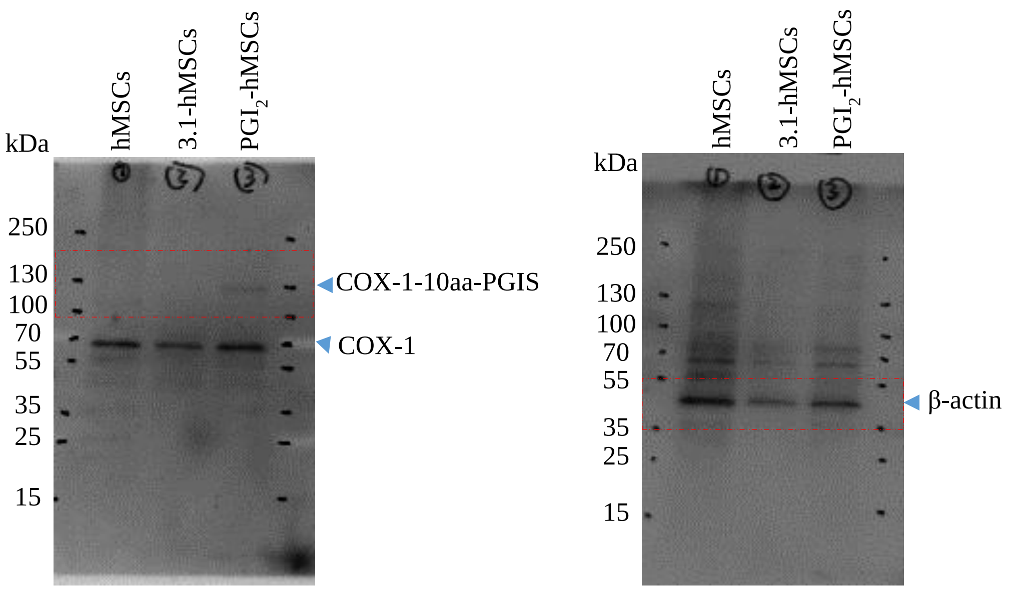

Supplementary Figure 5. Uncropped western blot images of Supplementary Figure1b. Cyclooxygenase-1 (COX-1) antibody detected the expression of the triple-catalytic enzyme (COX-1-10aa-PGIS) with a molecular mass of 130kDa in PGI2-hMSCs and the ubiquitously expressed form of COX-1 (70kDa) in hMSCs, 3.1-hMSCs, and PGI2-hMSCs.
